# Iterative Cross-Correlation Analysis of Resting State Functional Magnetic Resonance Imaging Data

**DOI:** 10.1371/journal.pone.0058653

**Published:** 2013-03-18

**Authors:** Liqin Yang, Fuchun Lin, Yan Zhou, Jianrong Xu, Chunshui Yu, Wen-Ju Pan, Hao Lei

**Affiliations:** 1 Wuhan Center for Magnetic Resonance, State Key Laboratory of Magnetic Resonance and Atomic and Molecular Physics, Wuhan Institute of Physics and Mathematics, Chinese Academy of Sciences, Wuhan, China; 2 Department of Radiology, RenJi Hospital, Jiao Tong University Medical School, Shanghai, China; 3 Department of Radiology, XuanWu Hospital, Capital Medical University, Beijing, China; 4 University of Chinese Academy of Sciences, Beijing, China; Oregon Health & Science University, United States of America

## Abstract

Seed-based cross-correlation analysis (sCCA) and independent component analysis have been widely employed to extract functional networks from the resting state functional magnetic resonance imaging data. However, the results of sCCA, in terms of both connectivity strength and network topology, can be sensitive to seed selection variations. ICA avoids the potential problems due to seed selection, but choosing which component(s) to represent the network of interest could be subjective and problematic. In this study, we proposed a seed-based iterative cross-correlation analysis (siCCA) method for resting state brain network analysis. The method was applied to extract default mode network (DMN) and stable task control network (STCN) in two independent datasets acquired from normal adults. Compared with the networks obtained by traditional sCCA and ICA, the resting state networks produced by siCCA were found to be highly stable and independent on seed selection. siCCA was used to analyze DMN in first-episode major depressive disorder (MDD) patients. It was found that, in the MDD patients, the volume of DMN negatively correlated with the patients' social disability screening schedule scores.

## Introduction

Resting state network (RSN) analysis utilizing the low frequency (<0.1 Hz) fluctuations (LFFs) in resting state functional magnetic resonance imaging (rs-fMRI) data has been extensively used for studying the intrinsic functional architectures of the brain [1], [2], [3], [4], [5], [6]. There are two commonly used methods for rs-fMRI data analysis: seed-based cross-correlation analysis (sCCA) [1] and independent component analysis [7]–[8]. The former measures the connectivity strength of brain voxels with a seed region-of-interest (ROI), and the latter mathematically decompose the LFFs into independent components, which are considered to represent different functional networks.

Methodologically both sCCA and ICA have some shortcomings. In traditional sCCA, the selection of seed ROI is, to some extent, subjective. It could be done based on either activated regions associated with specified tasks [1], [3], [4], [5], anatomically defined structures [6], or empirical coordinates from the literature [9]. Therefore the results of sCCA, in terms of both connectivity strength and network topology, may depend on the selection of seed ROI [4], [10].

ICA avoids the problem of seed ROI selection. However, choosing which component(s) to represent the network of interest could be problematic. The decision is not always straightforward, and may require strong a priori knowledge, especially for those networks that may be decomposed into more than one component [2], [11]. Template-matching procedures have been used for component identification, in which spatial correlations between the ICA components and a template of the network of interest were evaluated [12], [13]. However, this approach sometimes can be even less reliable than human rating [14]. Besides, in a way, selection of the template rises to be a new problem itself.

Here we propose a seed-based iterative cross-correlation analysis (siCCA) method for processing rs-fMRI data, with an expectation that the method will produce stable resting state brain networks, without puzzling over seed or component selection. The method was first applied to derive two well-established resting state brain networks in two independent datasets acquired from normal adults on a 3T scanner and a 1.5 T scanner, respectively. The robustness of siCCA was evaluated and compared to those of sCCA and group ICA. The siCCA method was also applied to a group of eighteen patients with first-episode major depressive disorder (MDD) to investigate the correlation between the properties of siCAA-derived resting state brain network and clinical assessment scores.

## Materials and Methods

### Ethics Statement

The study was approved by the Ethics Committee of Renji Hospital, Shanghai Jiaotong University School of Medicine and Xuanwu Hospital, Capital Medical University. The participants were informed of the aims of our study before MRI examinations. Full written informed consent was obtained from each participant.

### Subjects and data acquisition

Three groups of subjects were included in the study: 1) eighteen first-episode MDD patients, 2) eighteen normal adults with age and gender matched to the MDD patients, and 3) a third group of twenty normal adults. All subjects are right-handed Chinese adults. The normal subjects reported no history of neurological and psychiatric diseases. The MDD patients had not received any antidepressant treatment before MRI scan, and were assessed with Hamilton Depression Rating Scale (HDRS) [15], Hamilton Anxiety Rating Scale (HARS) [16] and Social Disability Screening Schedule (SDSS) [17] scores.

The resting state fMRI data for the first two groups of subjects were acquired on a 3.0 T Philips Achieva scanner in Renji Hospital, Shanghai Jiaotong University School of Medicine, Shanghai. The resting state fMRI data for the third group of subject were acquired on a 1.5 T Siemens Sonata scanner in Xuanwu Hospital, Capital Medical University, Beijing. Participants were instructed to lie quietly in the scanner during data acquisition, keeping motionless and their eyes closed but without falling asleep. A spin-echo single shot echo-planar pulse sequence was used to acquire the rs-fMRI data. Detailed demographic information of the subjects and the scanning parameters are listed in Table 1.

**Table 1:** Demographic information and scanning parameters of the three datasets.

Dataset	Philips 3.0T–NC	Philips 3.0T–MDD	Siemens 1.5T–NC
Age, range/mean (years)	21–49/30.1	22–30/29.9	23–34/25.6
Gender, M/F	6/12	6/12	6/14
TR/TE (ms)	2000/30	2000/30	2000/60
In-plane resolution	64×64	64×64	128×128
Field of view (mm^2^)	230×230	230×230	240×240
Slice thickness/gap (mm)	4/0	4/0	5/2
No. of axial slices per volume	34	34	20
No. of volumes	220	220	180

Abbreviation. NC: normal controls; MDD: major depressive disorder.

### Data Preprocessing

Data preprocessing were performed under the framework of statistical parametric mapping (SPM5, http://www.fil.ion.ucl.ac.uk/spm/). To avoid the effects of system instability and environmental adaptation, the first 10 volumes of the rs-fMRI data for each subject were discarded. The remaining volumes (i.e., 210 for Philips 3.0 T dataset and 170 for Siemens 1.5 T dataset) were put into subsequent processing procedures, including slice timing and head motion corrections, spatial normalization to the standard Montreal Neurological Institute (MNI) space (i.e., all data resampled to a voxel size of 3 mm×3 mm×3 mm), spatial smoothing with a 6-mm full width at half maximum (FWHM) isotropic Gaussian kernel, removal of linear drift and temporal band-pass filtering (0.01–0.08 Hz). No subjects were found to have head translation larger than 2 mm or rotation more than 2 degrees.

### sCCA

**Figure 1:**
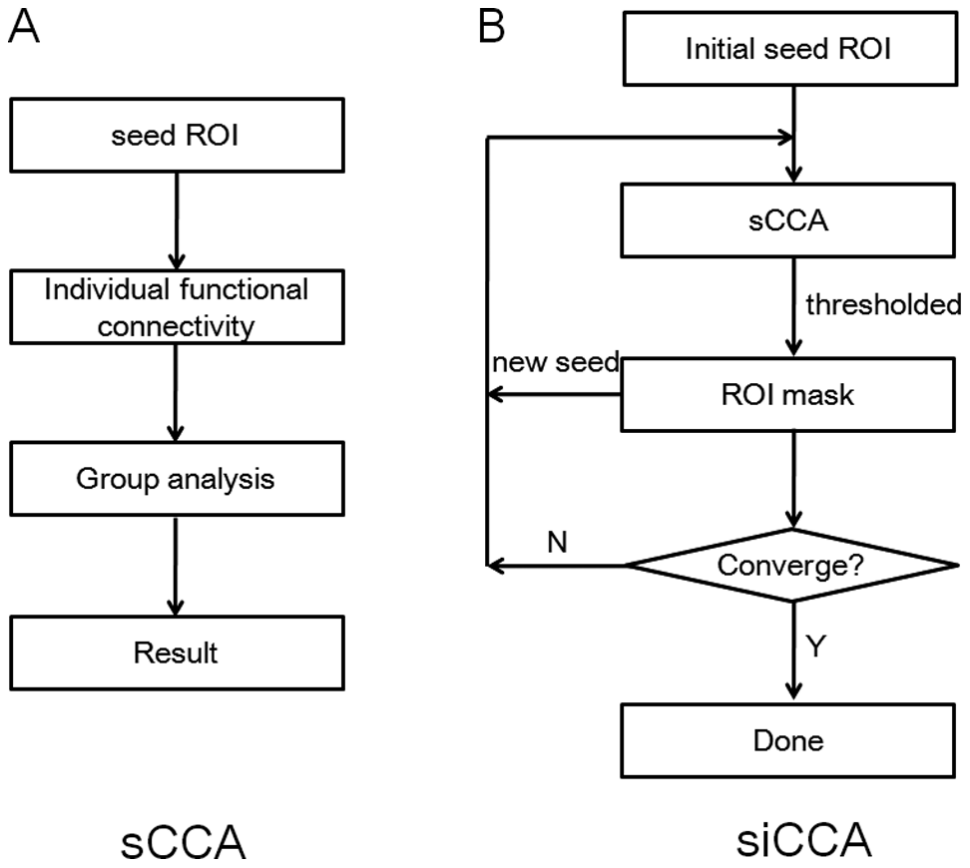
Flow charts of sCCA and siCCA.

The flowchart of sCCA is shown in Fig. 1A. Sources of spurious variance (i.e., head motion, global signal and signals from cerebrospinal fluid and white matter) were removed through linear regression [5]. After selection of an initial seed ROI, individual functional connectivity map was obtained by calculating the correlation coefficients between the time series of each brain voxel and the average time series of the seed ROI. A Fisher *r*-to-*z* transform was performed to increase the normality of the correlation coefficients [18]. Voxel-wise one-sample *t*-test was used to generate the group-level connectivity map at a threshold of *p*<0.001 (uncorrected) and cluster size >5.

### siCCA Analysis

The flowchart of siCCA is shown in Fig. 1B. After an initial seed ROI was selected and the correlation coefficient map for each individual was obtained, voxel-wise one-sample *t*-test was applied to generate the group-level connectivity map of the seed ROI at a threshold level of *p*<0.05 (corrected for family wise error) and voxel size>20. The ensemble of all the voxels in the resultant group-level connectivity map was then used as a new seed ROI for another round of sCCA. The iterative schedule was stopped if the results converge (i.e., the numbers of significant voxels obtained from two successive steps had a difference less than 10). This convergence threshold is quite strict as the iterative seed ROI typically consisting of more than 1000 voxels (more than 4000 voxels in the final ROI mask), resulting in above 99% overlap between ROI of two successive steps.

### Selection of Initial seed ROIs

Resting state networks analyzed here included the so-called default mode network (DMN) [19] and the stable task control network (STCN) [3]. A typical DMN includes posterior cingulate cortex (PCC)/precuneus region, medial prefrontal cortex (MPFC) and bilateral inferior parietal cortex (IPC) [2], [19]. Cerebellar regions, inferior temporal gyrus (ITG), parahippocampus gyrus (PHG), temporal poles, thalamus, orbital frontal cortex (OFC) and dorsolateral prefrontal cortex (DLPFC) were also reported to be part of the DMN [4], [5], [20]. The STCN is composed of anterior insula/frontal operculum (aI/fO), anterior prefrontal cortex (aPFC), dorsal anterior cingulate/medial superior frontal cortex (dACC/msFC) and thalamus [3].

The MNI coordinates of the seed ROIs used to derive DMN and STCN were obtained from the literature [3], [4], [5]. The seed ROIs for DMN included: 1) three concentric seeds in PCC with a center coordinate of [−5 −49 40] and a radius of 3, 6 and 9 mm respectively. These ROIs were thereafter denoted as PCC1-3, PCC1-6 and PCC1-9, respectively; 2) a 6-mm-radius seed in PCC with a center coordinate of [−12 −47 32] (thereafter denoted as PCC2-6); 3) a 6-mm-radius seed in mPFC with a center coordinate of [−1 47 −4] (thereafter denoted as MPFC-6). For STCN, four 6-mm-radius seed ROIs were selected from the right aI/fO [36, 16, 4], right aPFC [27, 50, 23], dACC/msFC [−1, 10, 46], and right anterior thalamus [10, −15, 8], respectively.

### ICA Analysis

Group spatial ICA was carried out using the Infomax algorithm [21] within the GIFT software (http://icatb.sourceforge.net/, version 1.3 h). The number of independent components was set to twenty [12]. A template-matching procedure was used to identify the components representing specific resting state networks [13]. In brief, a template of the resting state network of interest was first selected, with respect to which a “goodness-of-fit” score were calculated for each component from the group-average *z* map (i.e., subtracting the *z* score averaged across the voxels outside the template from that averaged across the voxels falling within the template). The component with the highest “goodness-of-fit” score was considered to reprensent the resting state network of interest. The template used to identify the ICA component(s) representing DMN was the group-level DMN derived using sCCA with PCC2-6 as the initial seed ROI (*p*<0.001, uncorrected and cluster size >5) [13]. For each component, a group-level *t* map was obtained with one sample *t*-test (*p*<0.001, uncorrected and cluster size>5). The group-level *t* maps of all the components were reviewed by human raters to identify the components that show similarity to the typical DMN reported in the literature [2], [3], [11].

### Evaluation of Network Similarity

To evaluate the similarities among the networks derived with different methods and seed ROIs, the intersection and union of the networks under concern were calculated. The number of the non-zero voxels in the intersection and that in the union were counted. The ratio between the two was defined as voxel-based similarity of networks (VBS_net_). VBS_net_ has a value between 0 and 1. The closer to 1 VBS_net_ is, the more similar the networks under concern are. In a way, VBS_net_ is similar to Jaccard similarity coefficient [22].

### siCCA-derived resting state brain networks in MDD patients

A group-level DMN (p<0.05, FWE corrected, cluster size>20) for the MDD patients was first obtained with the siCCA approach as described above. The initial seed ROI used was PCC2-6. The group-level result was then used as an ROI mask to extract DMN for each individual subject, which included those voxels whose time series had a correlation coefficient greater than 0.3 with the mean time series of voxels within the group-level DMN. Then the correlations between the volume (i.e., the number of voxels) of DMN and clinical assessments were investigated. All these results were compared with those obtained using the traditional sCCA approach to extract individual DMN.

## Results

### siCCA vs. sCCA

**Figure 2:**
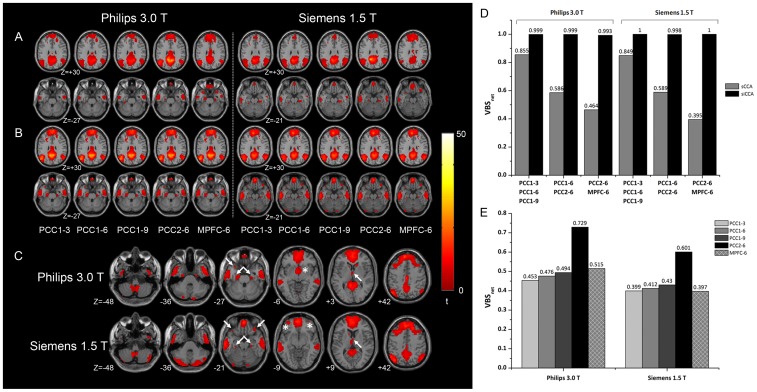
The default mode networks (DMN) derived from two datasets of normal adults groups using sCCA and siCCA. In (A) and (B), the DMNs obtained with different initial seed regions of interest (ROIs) using sCCA (A) and siCCA (B) are displayed in two axial slices (*z* = +30 and −27 for the Philips 3.0 T dataset; *z* = +30 and −21 for the Siemens 1.5 T dataset). The siCCA-derived DMNs are shown in additional axial slices in (C). White arrows and asterisks indicate the regions with different peak locations (PHG, temporal poles and thalamus) and distinct regions (a ACC/caudate/thalamus cluster for Philips 3.0 T and bilateral middle OFC for Siemens 1.5 T) in the two datasets respectively. The VBS_net_ index depicting the similarity among the DMNs obtained with different initial seed regions of interest was plotted in (D). The abscissa label in (D) describes the initial seed ROIs being compared. The similarity between the siCCA-derived DMN and the sCCA-derived DMNs with different initial seed ROIs was evaluated in (E). Please refer to the ***Selection of Initial seed ROIs*** section for definitions of the abbreviations for the seed ROIs.

Figure 2 shows the results regarding DMN derived from the two datasets. The sCCA-derived DMNs appeared to be influenced by the selection of initial seed ROI (Fig. 2A). Different aspects (i.e. radius, coordinates, and located brain regions) of the seed ROI, however, affected the results differentially (Fig. 2D). For both the two datasets, the DMNs derived from the concentric ROIs (PCC1-3, PCC1-6 and PCC1-9) had the least difference (VBS_net_ = 0.855 and 0.849 respectively), while those derived using seeds located in different brain regions (PCC2-6 vs. vmPFC-6) exhibited the largest difference (VBS_net_ = 0.464 and 0.395 respectively).

In comparison, for both datasets, the results of siCCA (Fig. 2B) remained stable (i.e., VSB_net_ score close to 1 as shown in Fig. 2D), regardless of how the seed ROI was selected. Additional slices of the siCCA results are shown in Fig. 2C. In both datasets, the siCCA-derived DMN included major brain regions considered to be part of typical DMN, such as PCC/precuneus, MPFC, IPC, cerebellum regions (i.e., tonsils and posterior lobes), ITG, PHG, temporal poles, thalamus and DLPFC (Fig. 2B and 2C). Some of the DMN structures within them, such as PHG, temporal poles and thalamus, however, had different peak locations in the two datasets (indicated by arrows in Fig. 2C). In addition, there were also differences between the DMN derived from the two datasets using siCCA. The siCCA-derived DMN using the Philips 3.0 T dataset included a cluster made up of ACC/caudate/thalamus (indicated by asterisk in Fig. 2C) similar with previous studies [4], which was not present in the Siemens 1.5 T results. On the other hand, presence of bilateral middle OFC in the DMN was found only for the 1.5 T dataset (indicated by asterisk in Fig. 2C), but not for the 3.0 T dataset.

The similarity between siCCA-derived DMN and the sCCA-derived DMN using different initial seed ROI was assessed (Fig. 2E). It was found that, for both datasets, the siCCA-derived DMN was most similar to the sCCA-derived network using PCC2-6 [4] as the initial seed ROI (VBS_net_ = 0.729 and 0.601 respectively) rather than using others (VBS_net_ smaller than 0.515 and 0.34 respectively).

**Figure 3:**
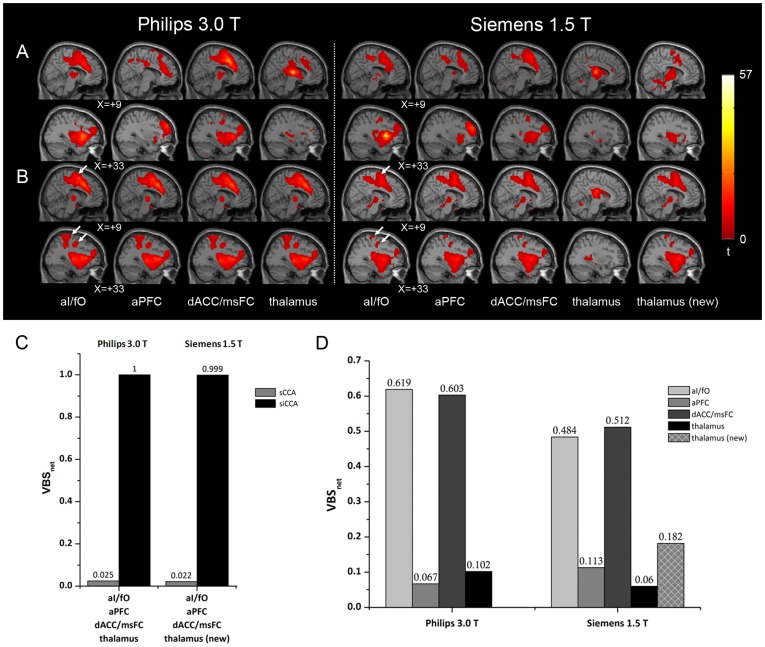
The stable task control networks (STCN*) derived from two datasets of normal adults groups using sCCA and siCCA. In (A) and (B), the STCN* obtained with different initial seed regions of interest (ROIs) using sCCA (A) and siCCA (B) are displayed in two saggital slices (*x* = +9 and +33). Please refer to the ***Selection of Initial seed ROIs*** section for definitions of the abbreviations for the seed ROIs. The seed ROI “thalamus” and the seed “thalamus (new)” have different center coordinates (i.e., [10, −15, 8] and [18, −9, 0] respectively). The former is obtained from the literature [3], and the latter is defined based on the siCCA-derived STCN* obtained from the Siemens 1.5 T dataset. White arrows in (B) indicate the regions (SMA and pre- and post-central gyrus) included in STCN* besides the four clusters analyzed. The VBS_net_ index depicting the similarity among the STCN* obtained with different initial seed regions of interest was plotted in (C). The abscissa label in (C) describes the initial seed ROIs being compared. The similarity between the siCCA-derived STCN* and the sCCA-derived STCN*with different initial seed ROIs was evaluated in (D).

Figure 3 shows the resting state functional networks derived with the seed ROIs considered to be part of STCN using sCCA (A) and siCCA (B), respectively. The similarity among the networks derived using different methods and different initial seed ROI was compared in Fig. 3C and D. For the Philips 3.0 T dataset, the sCCA-derived networks with different initial seed ROIs showed large difference (Fig. 3A and C). In contrast, the siCCA approach yielded stable results irrespective of which initial seed ROI was used (Fig. 3B and C). For the Siemens 1.5 T dataset, however, the use of seed ROI ‘thalamus’ resulted in networks obviously different from the networks derived with the other three initial seed ROIs, no matter whether sCCA or siCAA was used (Fig. 3B). It thus appeared that, for this particular dataset, the thalamus seed ROI selected based on literature results [3] was not part of the STCN. Given that the siCAA-derived networks using aI/fO, aPFC and dACC/msFC as the initial seed ROIs were stable (Fig. 3B and C), the peak location within the thalamic region of these networks [18, −9, 0] was used as the center coordinate of a new thalamus seed ROI (thereafter referred as thalamus (new)). The sCAA-derived network using thalamus [23] as the initial seed ROI showed improved resemblance to the traditional STCN, relative to the one using original thalamus seed ROI (i.e., center coordinate [10, −15, 8]). For both datasets, the seed-independent siCCA-derived network included not only all the main nodes considered to be part of the traditional STCN [3], but also other regions such as the supplementary motor cortex [24] and pre- and post-central gyrus (indicated by arrows in Fig. 3B). In order to distinguish these networks from the typical STCN reported by Dosenbach et al [3], they were thereafter referred as STCN*. The siCCA–derived STCN* showed more resemblance to the sCCA-derived networks using aI/fO and dACC/msFC as the initial seeds than to the sCCA-derived networks using aPFC or thalamus [23] as the initial seeds.

**Figure 4:**
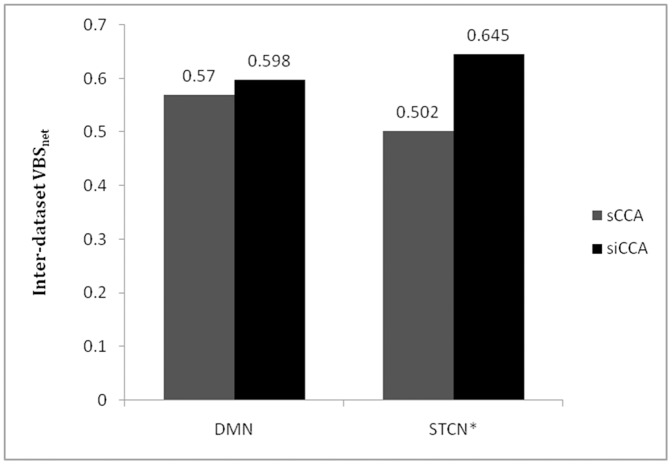
Similarity among the DMNs and STCN* obtained from two different datasets evaluated by inter-dataset VBS_net_. The networks being compared were derived using PCC2-6 and dACC/msFC for DMN and STCN* as the seed ROIs, respectively.

Inter-dataset VBS_net_ indices were also calculated to evaluate the consistency among the resting state networks derived from different datasets using sCCA and siCCA (Fig. 4). The networks compared were derived using PCC2-6 and dACC/msFC for DMN and STCN* as the seed ROIs, respectively. Relative to sCCA, siCCA improved the inter-dataset consistency of DMN only slightly (from 0.570 to 0.598), and that of STCN* moderately (from 0.502 to 0.645).

### siCCA vs. ICA

**Figure 5:**
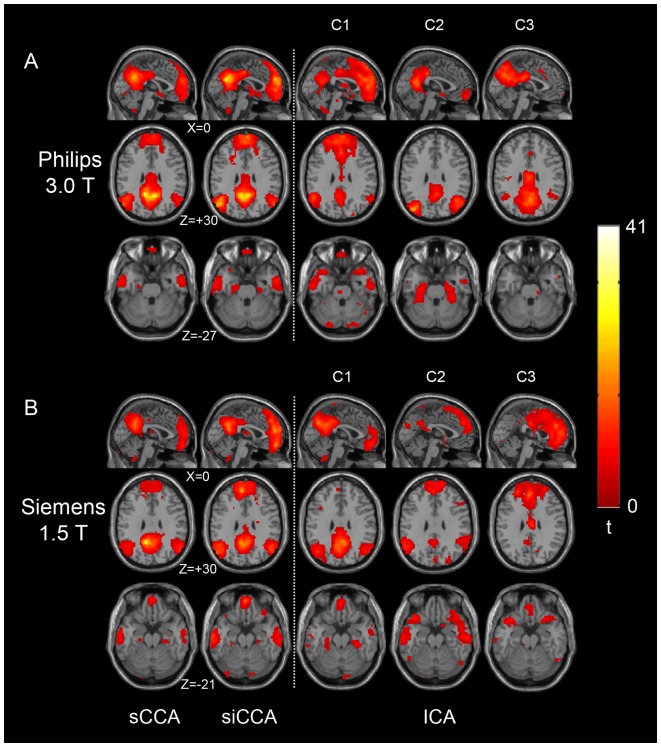
Comparison among the sCCA-, siCCA- and ICA-derived default mode networks from two datasets of normal adults groups. The sCCA- and siCCA-derived results with PCC2-6 as the initial seed ROI are displayed on the left side of the dashed line in one sagittal slice and two axial slices. The ICA-derived results are displayed on the right side of the dashed line. The three ICA components (C1, C2 and C3) with the highest ‘goodness-of-fit’ scores, with the sCAA-derived DMN as the template, are shown.

Figure 5 compares the DMNs obtained with sCCA, siCCA and ICA. The results of sCAA and siCAA were derived using PCC2-6 as the initial seed ROI. Using the sCAA-derived DMN as the template, the first three ICA components with the highest “goodness-of-fit” scores (i.e., C1, C2 and C3) were selected and presented. For both datasets, the DMNs appeared to have been decomposed into more than one component. For the Philips 3.0 T dataset, C1 was mainly composed of the anterior area of DMN while C2 and C3 constitute the posterior area of DMN as reported in previous studies [2], [11]. For the Siemens 1.5 T dataset, on the other hand, C1 was mainly composed of the posterior area of DMN, while the anterior parts were decomposed into C2 and C3.

### DMN of MDD patients extracted by siCCA

**Figure 6:**
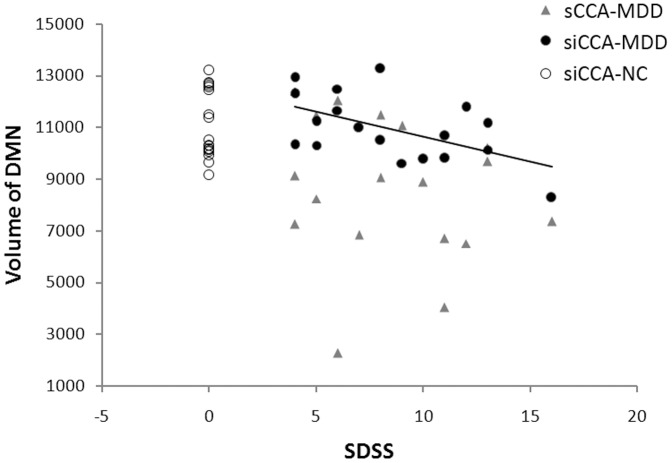
Correlation between the volume of siCCA-derived DMN and SDSS score in MDD patients. DMN in the MDD patients and matched normal controls were derived using siCAA and sCAA with PCC2-6 as the initial seed. For the MDD patients, the average volume of siCAA-derived DMN (*black dots*) was significant larger than that derived with sCCA (*gray triangles*). However, the volume of siCAA-derived DMN was not significantly different between the MDD patients and normal controls (*black circles*). For the MDD patients, a significant correlation was found between the volume of siCCA-derived DMN and SDSS score (*solid line*: r = −0.545, *p* = 0.019).

The clinical assessments for the MDD patients were: HDRS 23.6±4.7, HARS 19.2±6.6, and SDSS 8.4±3.6. The DMN volumes of MDD patients derived with siCCA were significant larger than that derived with sCCA (*p* = 0.002, two-sample *t* test, Fig. 6). No significant difference was found between siCCA-derived DMN volumes in MDD patients and those in matched normal controls (i.e. NC group). Correlation analysis revealed that, a significant negative correlation existed between the volume of siCCA-derived DMN and SDSS score in MDD patients (r = −0.545, *p* = 0.019). In contrast, the sCCA-derived DMN did not show similar correlation (r = −0.195, *p* = 0.349).

## Discussion

To extract a resting state network of interest, the traditional sCCA approach first calculates correlation coefficients between the time courses of individual brain voxel and the mean time course of an initial seed ROI for each subject. Statistical thresholding was then applied to yield a group-level network. The resting state networks derived by sCCA may depend on how the initial seed is selected, demonstrated both by our results and previous studies [4], [10]. One reason for this is that the mean time course of the initial seed ROI contains not only features characterizing the resting state network of interest, but also features specific to the seed ROI itself or the brain region it locates in. The seed ROI-specific signals may come from the following sources: 1) within a given resting state network, some nodes may serve as hubs characterized by having direct connections to nearly all the other network nodes, while the others may have only indirect connections among each other. The sCCA-derived resting networks would thus likely be affected by whether the seed-ROI is positioned in a hub brain region or in a non-hub brain region, 2) some brain regions may participate in more than one resting state networks that overlap with each other, making its time series a combined result [25]. If the initial seed is positioned in such regions, the correlation pattern of the seed would reflect all contributing networks rather than only the one we are interested, 3) the mean time course of the seed-ROI includes contributions from random background noises, and 4) cross-subjection variations in connectivity may be different for different nodes in a given resting state network. Positioning the seed ROI in a brain region with less cross-subjection variations in connectivity would likely result in more consistent network.

The initial steps in siCAA were exactly the same as those in sCCA, namely calculating the resting state network connecting to the initial seed ROI for each subject and statistical thresholding to yield a group-level network (Fig. 1). Instead of being considered as the final result as in sCCA, this group-level network was used as a new seed ROI to perform the next round of sCCA analysis in siCCA. Starting from this point, the reference time course for correlation analyses no longer represent merely the features of the initial seed ROI, but rather the features of a network with the initial seed ROI as one of its nodes. Depending on the exact architecture of the network of interest, the reference time course may be biased towards the contributions from larger or hub nodes of the network, while the contributions from the signals specific to the seed ROI initially selected were diminished. This may explain why the siCCA-derived resting state networks are stable and seed-independent, as long as the initial seed ROIs selected were within the resting state network of interest (Figs. 2 and 3). This is supported by the observation that sCCA using major/hub nodes (such as PCC2 in DMN, and aI/fO and dACC/msFC in STCN*) within the network of interest as the initial seed ROIs yielded results more resemble to those obtained by siCCA.

There are other mechanisms may have also contributed to the stability of the siCCAs. For example, the seed ROI in siCCA (i.e., starting from the second round of iteration) is made of a group-level network that may have a much larger size (i.e., in terms of the number of voxels) than the initial seed ROI selected. A physically larger seed ROI may have a mean time course with higher signal-to-noise ratio (SNR), and thus less effect from the background noises. Moreover, repeated group-level statistical thresholding in siCCA (Fig. 1B) makes only the voxels with low cross-subject variations of connectivity properties (i.e., stable at the group level) were included in the resultant network. The final result of siCCA thus represents the connectivities of the brain voxels to a refined network with the initial seed ROI as one of its nodes.

The most important parameter in siCCA is the statistical threshold for generating the group-level network that could be subsequently used as the *new* seed ROI for the next round of correlation analyses. In this study, we optimized this parameter empirically to a threshold level of *p*<0.05, corrected for family-wise error and a cluster size>20. It can be shown that a more lenient threshold would produce more distributed networks, with an extreme that all brain voxels being classified into two anti-correlated functional networks [5]. A tighter threshold would reduce the extent of the network, with an extreme that only the local connectivities to the initial seed ROI are revealed.

The use of the siCCA approach improved the cross-dataset consistency between the STCN* obtained from the 1.5 T dataset and that from the 3 T dataset moderately, but not the cross-dataset consistency between the DMNs obtained from the two datasets (Fig. 4). This is in accordance to the known fact that the resting state networks may differ among different populations [2], [9], and the extent of cross-subject variations are different for different resting state networks [2].

The DMN is thought to be related to a series of internal self-referential brain processes [26], [27] and accounts for person's individual variability such as task performance [28]. A study in autism found a correlation between DMN region's activity and clinical social impairment scores [29], suggesting that the social interaction ability was related to DMN. Previous resting state studies of MDD patients' brain had revealed a lot of altered connectivity between regions or regions and networks, and several results were correlated with clinical symptoms (see ref [30] for review). In this study, we focus on the basic overall volume information of DMN rather than the connections. According to the correlation analysis result, smaller resting state DMN volume of MDD patients correspond to higher severity of social disability. As discussed above, a smaller DMN may represents a concentrated, autistic ‘self’ that segregated from external milieu or other brain functions. This correlation may reflect a MDD-based brain dysfunction as the MDD's group DMN extracted with siCCA was used, or reflect subject's personality features as no significant differences were found between DMN and NC group. Either of them reveals that siCCA could produce more credible information about the internal functional architecture than traditional sCCA.

In conclusion, the siCCA approach we proposed here is a reliable method for extraction of resting state networks. The siCCA-derived resting state networks are stable and seed-independent, as long as the initial seed ROIs selected were within the resting state network of interest. Inter-group comparison of the siCCA-derived resting state networks can be performed in a way similar to what have been done for ICA-derived resting state networks [31], but without puzzling over how to select the proper component(s) to represent the network of interest. It was demonstrated in the MDD patients that the property of siCCA-derived resting state networks, such as the volume of DMN, correlated better with the clinical assessment, than that of the ones derived with sCCA.

## References

[1] Biswal B, Yetkin FZ, Haughton VM, Hyde JS (1995) Functional connectivity in the motor cortex of resting human brain using echo-planar MRI. Magnetic Resonance in Medicine 34: 537–541. 8524021 10.1002/mrm.1910340409

[2] Damoiseaux JS, Rombouts S, Barkhof F, Scheltens P, Stam CJ, et al (2006) Consistent resting-state networks across healthy subjects. Proceedings of the National Academy of Sciences of the United States of America 103: 13848–13853. 16945915 10.1073/pnas.0601417103 PMC1564249

[3] Dosenbach NUF, Fair DA, Miezin FM, Cohen AL, Wenger KK, et al (2007) Distinct brain networks for adaptive and stable task control in humans. Proceedings of the National Academy of Sciences of the United States of America 104: 11073–11078. 17576922 10.1073/pnas.0704320104 PMC1904171

[4] Greicius MD, Krasnow B, Reiss AL, Menon V (2003) Functional connectivity in the resting brain: A network analysis of the default mode hypothesis. Proceedings of the National Academy of Sciences of the United States of America 100: 253–258. 12506194 10.1073/pnas.0135058100 PMC140943

[5] Fox MD, Snyder AZ, Vincent JL, Corbetta M, Van Essen DC, et al (2005) The human brain is intrinsically organized into dynamic, anticorrelated functional networks. Proceedings of the National Academy of Sciences of the United States of America 102: 9673–9678. 15976020 10.1073/pnas.0504136102 PMC1157105

[6] Lowe MJ, Mock BJ, Sorenson JA (1998) Functional connectivity in single and multislice echoplanar imaging using resting-state fluctuations. Neuroimage 7: 119–132. 9558644 10.1006/nimg.1997.0315

[7] Steriade M, Contreras D, Amzica F (1994) SYNCHRONIZED SLEEP OSCILLATIONS AND THEIR PAROXYSMAL DEVELOPMENTS. Trends in Neurosciences 17: 199–208. 7520202 10.1016/0166-2236(94)90105-8

[8] McKeown MJ, Makeig S, Brown GG, Jung TP, Kindermann SS, et al (1998) Analysis of fMRI data by blind separation into independent spatial components. Human Brain Mapping 6: 160–188. 9673671 10.1002/(SICI)1097-0193(1998)6:3<160::AID-HBM5>3.0.CO;2-1 PMC6873377

[9] Long XY, Zuo XN, Kiviniemi V, Yang Y, Zou QH, et al (2008) Default mode network as revealed with multiple methods for resting-state functional MRI analysis. Journal of Neuroscience Methods 171: 349–355. 18486233 10.1016/j.jneumeth.2008.03.021

[10] Ma LS, Wang BQ, Chen XY, Xiong JH (2007) Detecting functional connectivity in the resting brain: a comparison between ICA and CCA. Magnetic Resonance Imaging 25: 47–56. 17222714 10.1016/j.mri.2006.09.032

[11] Damoiseaux JS, Beckmann CF, Arigita EJS, Barkhof F, Scheltens P, et al (2008) Reduced resting-state brain activity in the “default network” in normal aging. Cerebral Cortex 18: 1856–1864. 18063564 10.1093/cercor/bhm207

[12] Garrity AG, Pearlson GD, McKiernan K, Lloyd D, Kiehl KA, et al (2007) Aberrant “default mode” functional connectivity in schizophrenia. American Journal of Psychiatry 164: 450–457. 17329470 10.1176/ajp.2007.164.3.450

[13] Greicius MD, Srivastava G, Reiss AL, Menon V (2004) Default-mode network activity distinguishes Alzheimer's disease from healthy aging: Evidence from functional MRI. Proceedings of the National Academy of Sciences of the United States of America 101: 4637–4642. 15070770 10.1073/pnas.0308627101 PMC384799

[14] Franco AR, Pritchard A, Calhoun VD, Mayer AR (2009) Interrater and Intermethod Reliability of Default Mode Network Selection. Human Brain Mapping 30: 2293–2303. 19206103 10.1002/hbm.20668 PMC2751639

[15] Hamilton M (1967) Development of a rating scale for primary depressive illness. British Journal of Social and Clinical Psychology 6: 278–&. 6080235 10.1111/j.2044-8260.1967.tb00530.x

[16] Hamilton M (1959) The assessment of anxiety states by rating. The British journal of medical psychology 32: 50–55. 13638508 10.1111/j.2044-8341.1959.tb00467.x

[17] Shen YWC (1985) A Handbook of Epidemiological Investigation of Mental Illness.

[18] Press WH, Teukolsky SA, Vetterling WT, Flannery BP (1992) Numerical Recipes in C (second ed.). 637 p.

[19] Raichle ME, MacLeod AM, Snyder AZ, Powers WJ, Gusnard DA, et al (2001) A default mode of brain function. Proceedings of the National Academy of Sciences of the United States of America 98: 676–682. 11209064 10.1073/pnas.98.2.676 PMC14647

[20] Fransson P (2005) Spontaneous low-frequency BOLD signal fluctuations: An fMRI investigation of the resting-state default mode of brain function hypothesis. Human Brain Mapping 26: 15–29. 15852468 10.1002/hbm.20113 PMC6871700

[21] Bell AJ, Sejnowski TJ (1995) An information maximization approach to blind separation and blind deconvolution. Neural Computation 7: 1129–1159. 7584893 10.1162/neco.1995.7.6.1129

[22] Jaccard P (1901) Étude comparative de la distribution florale dans une portion des Alpes et des Jura. Bulletin de la Société Vaudoise des Sciences Naturelles 37: 547–579.

[23] Rogers BP, Morgan VL, Newton AT, Gore JC (2007) Assessing functional connectivity in the human brain by fMRI. Magnetic Resonance Imaging 25: 1347–1357. 17499467 10.1016/j.mri.2007.03.007 PMC2169499

[24] Weissman MM, Bland RC, Canino GJ, Faravelli C, Greenwald S, et al (1996) Cross-national epidemiology of major depression and bipolar disorder. Jama-Journal of the American Medical Association 276: 293–299. 8656541

[25] Smith SM, Miller KL, Moeller S, Xu JQ, Auerbach EJ, et al (2012) Temporally-independent functional modes of spontaneous brain activity. Proceedings of the National Academy of Sciences of the United States of America 109: 3131–3136. 22323591 10.1073/pnas.1121329109 PMC3286957

[26] Gusnard DA, Akbudak E, Shulman GL, Raichle ME (2001) Medial prefrontal cortex and self-referential mental activity: Relation to a default mode of brain function. Proceedings of the National Academy of Sciences of the United States of America 98: 4259–4264. 11259662 10.1073/pnas.071043098 PMC31213

[27] Buckner RL, Andrews-Hanna JR, Schacter DL (2008) The brain's default network: Anatomy, function, and relevance to disease. Ann NY Acad Sci 1124: 1–38. 18400922 10.1196/annals.1440.011

[28] Hampson M, Driesen NRS, P G, J C, Constable RT (2006) Brain connectivity related to working memory performance. J Neurosci 26: 13338–13343. 17182784 10.1523/JNEUROSCI.3408-06.2006 PMC2677699

[29] Kennedy DP, Redcay E, ourchesne E (2006) Failing to deactivate: resting functional abnormalities in autism. Proc Natl Acad Sci USA 103: 8275–8280. 16702548 10.1073/pnas.0600674103 PMC1472462

[30] Wang L, Hermens DF, Hickie IB, Lagopoulos J (2012) A systematic review of resting-state functional-MRI studies in major depression. Journal of Affective Disorders 142: 6–12. 22858266 10.1016/j.jad.2012.04.013

[31] Greicius MD, Flores BH, Menon V, Glover GH, Solvason HB, et al (2007) Resting-state functional connectivity in major depression: Abnormally increased contributions from subgenual cingulate cortex and thalamus. Biological Psychiatry 62: 429–437. 17210143 10.1016/j.biopsych.2006.09.020 PMC2001244

